# Supplementary material for: A cautionary note on the use of unsupervised machine learning algorithms to characterise malaria parasite population structure from genetic distance matrices
Source: PLoS Genet. 2020 Oct 9;16(10):e1009037. doi: 10.1371/journal.pgen.1009037 (PMC7577480; doi:10.1371/journal.pgen.1009037)
Supplement: S1 Text — (PDF) [file pgen.1009037.s001.pdf]

S1 Text. A note on the use of IBD for inter-population comparisons and the construction of molecular barcodes.

We added this supplementary note following a reviewer comment, summarised as:

- How can the distance measure 1-IBD be used for inter-population comparisons, and
- Can 1-IBD be used to identify genetic regions that are driving population structure (useful for those working on molecular barcodes for geographical and transmission classification)

Considering definitions of IBD, this comment touches upon two different points. First, how to transition between inter-individual measures of IBD to inter-population measures. Second, the difference between a per-locus measure of IBD (e.g. IBD segments as reported in [1]) and a genome-wide measure of IBD (e.g. relatedness as defined in [5]). hmmIBD [6] and isoRelate [1,2] both generate estimates of IBD segments from the most likely sequence of IBD states; hmmIBD also outputs an estimate of relatedness ("fract_sites_IBD"), while isoRelate could easily be adapted to do so (last time we checked it did not, however). Moreover, it is possible to average over a per-locus IBD measure to generate a genome-wide IBD measure. However, this is not always ideal, especially when the data are limited. For example, a genome-wide measure of relatedness averaged over the most likely sequence of IBD states does not account for all possible sequences of IBD states, which is problematic because the most likely sequence, despite being the most likely, has a very low probability of being the true sequence [3].

Molecular barcodes provide adequate data to estimate inter-individual probabilities of IBD at any position over the genome (e.g. genome-wide measures of relatedness as defined in [5]). They do not provide adequate data to estimate accurately the probability of inter-individual IBD at specified positions over the genome (e.g. IBD segments as reported in [1]). (Aside: whole genome sequence data are required to estimate per-locus measures of inter-individual IBD; these can then be converted into per-locus measures of inter-population IBD, e.g. by averaging over individuals as in [1]) As such, IBD estimates generated from molecular barcode data are not able to identify genetic regions that are driving population structure (e.g. we cannot use molecular barcodes to re-create Fig 4 of [1]). However, they could be used to e.g. monitor changes in transmission (we do not know of an example yet where IBD measures from barcodes have been used to do this; although genome-wide measures derived from WGS data have [4]), and connectivity between locations on a population level (e.g. [7,8]). For inter-population analyses, one must convert many inter-individual IBD measures into inter-population IBD measures. To date, studies have done this in a variety of ways. For example, by calculating the fraction of inter-individual IBD measures that exceed some threshold (e.g. the fraction whose relatedness is greater than 0.5 [7]). More recently, approaches from optimal transport have been used to in a threshold-free manner that essentially amounts to calculating the cost of transporting a distribution of parasites samples from population to another [8]. There is still a need for a more theoretical analysis of which approach is optimal and in what setting.

References

[1] Henden L, Lee S, Mueller I, Barry A, Bahlo M. Identity-by-descent analyses for measuring population dynamics and selection in recombining pathogens. PLoS genetics. 2018;14(5):e1007279.

[2] Henden L, Wakeham D, Bahlo M. XIBD: software for inferring pairwise identity by descent on the X chromosome. Bioinformatics. 2016;32(15):2389–2391. doi:10.1093/bioinformatics/btw124.

[3] Rabiner LR. A tutorial on hidden Markov models and selected applications in speech recognition. Proceedings of the IEEE. 1989;77(2):257–286.

[4] Daniels RF, Schaffner SF, Wenger EA, Proctor JL, Chang HH, Wong W, et al. Modeling malaria genomics reveals transmission decline and rebound in Senegal. Proceedings of the National Academy of Sciences. 2015;112(22):7067–7072.

[5] Taylor AR, Jacob PE, Neafsey DE, Buckee CO. Estimating relatedness between malaria parasites. Genetics. 2019; p. genetics–302120.

[6] Schaffner SF, Taylor AR, Wong W, Wirth DF, Neafsey DE. hmmIBD: software to infer pairwise identity by descent between haploid genotypes. Malaria Journal. 2018;17(1):196.

[7] Taylor AR, Schaffner SF, Cerqueira GC, Nkhoma SC, Anderson TJ, Sriprawat K, et al. Quantifying connectivity between local *Plasmodium falciparum* malaria parasite populations using identity by descent. PLoS Genetics. 2017;13(10):e1007065.

[8] Taylor AR, Echeverry DF, Anderson TJC, Neafsey DE, Buckee CO. Identity-by-descent relatedness estimates with uncertainty characterise departure from isolation-by-distance between *Plasmodium falciparum* populations on the Colombian-Pacific coast. [Preprint] bioRxiv. 2020;doi:10.1101/2020.04.10.035303.
