# Supplementary material for: A cautionary note on the use of unsupervised machine learning algorithms to characterise malaria parasite population structure from genetic distance matrices
Source: PLoS Genet. 2020 Oct 9;16(10):e1009037. doi: 10.1371/journal.pgen.1009037 (PMC7577480; doi:10.1371/journal.pgen.1009037)
Supplement: Attachment — Submitted filename: PCoA_HAC_AAN.docx [file pgen.1009037.s002.docx]

The authors have identified a long-standing important problem in malaria population genetics and genetic epidemiology research; the determination of ancestral links between isolates within and between geographic and temporal populations. Ancestry (the full ancestral recombination graph) is fundamental to most questions on transmission flux and dynamics as well as determining the evolution of important phenomena such as drug resistance and the consequent selective sweeps. The authors have used a clear example of a sympatric population of *Plasmodium falciparum* from the Greater Mekong region (the epicentre of antimalarial resistance emergence), to demonstrate the problems with the approaches that are most commonly used to define the underlying structure, PCoA and HAC. These they referred to as Machine learning (ML) approaches, though it would be more precise to refer to these as unsupervised machine learning and limit the use of the ML abbreviation, which could be mis-understood for Maximum Likelihood. They clearly introduced the problem and reduced this to two major issues;

1. Data dimension reduction
2. Cluster determination and visualisation

There are a variety of data reduction approaches for which PCA/PCoA are one and these mostly go through a distance/similarity matrix. In this case, genetic polymorphisms are collapsed into an arbitrary or a model-based distance matrix used for determining higher dimension in the data structure. I agree with the authors that projecting the dimensions from these approaches and finding clusters cannot be interpreted directly as if each cluster is defined by a unique ancestral lineage. I however believe that this caution applies more when the underlying distance matrix does not take into consideration evolutionary events (mutation, recombination and linkage) that have defined the genetic data structure. Several datatypes (especially SNPs and Microsatellites) are used for these analyses. Most often these data are trimmed to reduce linkage and for microsatellites there is an assumption of neutrality that may be inaccurate. The authors have not elaborated on how distance matrices from these whole genome or subset of data types may affect the outcomes and inferences.

As the authors stated, methods such as chromopainter and fineStructure take into account recombination and mutation rates in modelling distances. However, this was not the focus of subsequent proof of principle against these approaches. It would have helped to include matrices from chromopainter and Relate to compare with IBD and IBS distance measures in reconstructing of the true ancestral tree of a population . Therefore, the statement that model-based approaches applied in Structure, fineStructure and Admixture tools only ‘loosely’ apply to malaria parasites was not proven here as these were not compared with the IBD, IBS methods tested. All three model-based approaches have been tested in humans and have been shown to robustly discern human history. It is true that their application on malaria parasites could be complicated by transmission through vector, sexual recombination and selfing, requiring further sensitivity testing before conclusive inferences. Recombination also occurs in human populations while ‘inbreeding’ in some ethnic populations to some degree is close to ‘selfing’. Selfing results in highly identical genomes and these can be weeded out prior to structure analysis using model-based approaches. Reference 6 indeed used chromopainter ancestry matrix and therefore testing the sensitivity of this matrix to the downstream clustering algorithm and parameters could have further helped with clarity on future directions.

To show the limitations of PCoA, the authors used a selected set of monoclonal samples from the TRAC study. By selecting only monoclonals, the authors avoided and important problem equally needing solution for malaria population genetics and genetic epidemiology, multiclonality and how these are related. The subsequent PCoA analysis with the different distance measures clearly showed the limits of these approach as the -log2IBD did not reproduced the previously published results. I noted that authors applied an approximation for -log2IBD when IBD is 0. The assumption here is that this approximation has no effect. As it was still possible to interpret PC1 of the -log2IBD plot, this to me remains useful for exploration against an underlying hypothesis.

The isolates employed were sympatric and therefore connected or share a recent common ancestor. It is therefore more cautious to interpret PCoA when populations are connected and recombining. Further clarity would have been gained if these three distances were tested for sensitivity against a simulated population with known ancestral recombination graph.

I agree with the authors on the arguments against HAC and use of heatmaps for visualisation though these as well were not tested for sensitivity of the seriation approach on distinguishing known clusters (ancestral groups).

Minor issues

- Figure 4 is clearly illustrative of issues with HAC but it is the branch heights that matter and not the horizontal axis which is an arbitrary arrangement.
- Can agglomerative coefficient be used to increase the confidence in HAC
- Testing of HAC as shown in figure 6 gives the impression that the average linking algorithm is best as the stability of its clusters were tested using the other methods. This misinterpretation will remain if either of the other linkage functions were first used.
- The authors gave little explanation on the outputs from figure 7
- Why did they target only monoclonal isolates? What will happen of populations are highly polyclonal as is the case in some settings in Africa?
- The steps before getting the data to use for genetic distance calculation shape the structure of the final set. Recommendations should begin from here
- What are the scientific questions that can be answered by PCoA and HAC?
- What are the inferential methods that the 2 approaches can be replaced with?
- No proposition of statistical models that can bridge the disconnect they have shown
- The authors are recommending approaches such as implemented in Relate. How will the problem of differences in recombination rates between populations with different transmission intensities be resolved? While this was not part of the investigation, a recommendation will be useful to the malaria popgen community.
- Emphasis is placed on transparency by making public the distance matrices used. This is an excellent recommendation though it will not resolve issues with published data but rather allow for reproduction or verification

Overall, the paper is timely and highly relevant to the malaria population genetics community.
