# Supplementary material for: A cautionary note on the use of unsupervised machine learning algorithms to characterise malaria parasite population structure from genetic distance matrices
Source: PLoS Genet. 2020 Oct 9;16(10):e1009037. doi: 10.1371/journal.pgen.1009037 (PMC7577480; doi:10.1371/journal.pgen.1009037)
Supplement: Attachment — Submitted filename: Reviewer_responses_final.docx [file pgen.1009037.s003.docx]

Reviewer 1

The authors have identified a long-standing important problem in malaria population genetics and genetic epidemiology research; the determination of ancestral links between isolates within and between geographic and temporal populations. Ancestry (the full ancestral recombination graph) is fundamental to most questions on transmission flux and dynamics as well as determining the evolution of important phenomena such as drug resistance and the consequent selective sweeps. The authors have used a clear example of a sympatric population of *Plasmodium falciparum* from the Greater Mekong region (the epicentre of antimalarial resistance emergence), to demonstrate the problems with the approaches that are most commonly used to define the underlying structure, PCoA and HAC. These they referred to as Machine learning (ML) approaches, though it would be more precise to refer to these as unsupervised machine learning and limit the use of the ML abbreviation, which could be mis-understood for Maximum Likelihood.

We thank the reviewer for these comments and for taking time to read our manuscript in detail. We agree that “unsupervised machine learning” is more precise and we have changed this in the title and the main text. The ML abbreviation has been removed entirely.

They clearly introduced the problem and reduced this to two major issues:

1. Data dimension reduction
2. Cluster determination and visualisation

There are a variety of data reduction approaches for which PCA/PCoA are one and these mostly go through a distance/similarity matrix. In this case, genetic polymorphisms are collapsed into an arbitrary or a model-based distance matrix used for determining higher dimension in the data structure. I agree with the authors that projecting the dimensions from these approaches and finding clusters cannot be interpreted directly as if each cluster is defined by a unique ancestral lineage. I however believe that this caution applies more when the underlying distance matrix does not take into consideration evolutionary events (mutation, recombination and linkage) that have defined the genetic data structure. Several datatypes (especially SNPs and Microsatellites) are used for these analyses. Most often these data are trimmed to reduce linkage and for microsatellites there is an assumption of neutrality that may be inaccurate. The authors have not elaborated on how distance matrices from these whole genome or subset of data types may affect the outcomes and inferences.

We agree that this is an important aspect. Interpreting the output of a statistical method that takes as input a generic distance matrix necessitates first of all being able to interpret the distance measure used to construct the matrix. Data pre-processing choices will affect the interpretation of the distance measure. However, even when the distance is interpretable and considers recent evolutionary events such as recombination (for example the 1-relatedness distance measure), it is unclear how exactly to interpret the output of PCoA and HAC (the coalescent model assumptions used in the phylogenetic interpretation of PCA do not hold for PCoA). We refer the reader to an in-depth analysis of distance measures (Taylor et al, Genetics 2019: reference 12 added on line 434).

*“The genetic distances that feature within the distance matrix can be either computed directly as a function of the genetic data (e.g. the fraction of loci where observed alleles differ) or inferred under a statistical model (e.g. the model underpinning chromosome painting [4]). The distance tends to be more interpretable when inferred under a statistical model that references a particular evolutionary process [12], however the inferential process is more involved, and thus possibly less transparent.”*

As the authors stated, methods such as chromopainter and fineStructure take into account recombination and mutation rates in modelling distances. However, this was not the focus of subsequent proof of principle against these approaches. It would have helped to include matrices from chromopainter and Relate to compare with IBD and IBS distance measures in reconstructing of the true ancestral tree of a population. Therefore, the statement that model-based approaches applied in Structure, fineStructure and Admixture tools only ‘loosely’ apply to malaria parasites was not proven here as these were not compared with the IBD, IBS methods tested. All three model-based approaches have been tested in humans and have been shown to robustly discern human history. It is true that their application on malaria parasites could be complicated by transmission through vector, sexual recombination and selfing, requiring further sensitivity testing before conclusive inferences. Recombination also occurs in human populations while ‘inbreeding’ in some ethnic populations to some degree is close to ‘selfing’. Selfing results in highly identical genomes and these can be weeded out prior to structure analysis using model-based approaches. Reference 6 indeed used chromopainter ancestry matrix and therefore testing the sensitivity of this matrix to the downstream clustering algorithm and parameters could have further helped with clarity on future directions.

We thank the reviewer for this comment and agree that the wording was misleading.

We have changed the text in the introduction (starting line 58):

*“Their empirical success in human genetics does not directly imply that they are applicable to population genetic analyses of malaria parasites [...]. There is a need to test the sensitivity of these methods in the context of malaria parasite genetics before making conclusive inferences.”*

For completeness we have added chromosome painting and a PCoA of the output co-ancestry matrix as an additional analysis. Fig 2 now shows PCoA of the 3 distance matrices and PCoA of the co-ancestry matrix.

To show the limitations of PCoA, the authors used a selected set of monoclonal samples from the TRAC study. By selecting only monoclonals, the authors avoided and important problem equally needing solution for malaria population genetics and genetic epidemiology, multiclonality and how these are related. The subsequent PCoA analysis with the different distance measures clearly showed the limits of these approach as the -log2IBD did not reproduce the previously published results. I noted that authors applied an approximation for -log2IBD when IBD is 0. The assumption here is that this approximation has no effect. As it was still possible to interpret PC1 of the -log2IBD plot, this to me remains useful for exploration against an underlying hypothesis.

We agree that we avoided a key problem but we also argue that this is outside of the scope of this paper. What to do with polyclonal samples is often swept under the rug in applied analyses. We have made the following changes to highlight its importance:

Fig 1 (Showing the computational analysis pipeline) now has an extra step which is the data pre-processing. Two of the examples of pre-processing steps are now: “*Remove polyclonal samples based on an estimate of within host diversity*” and *“Phase polyclonal samples (e.g. deploidIBD)”.*

We have added in the Introduction (starting line 111):

*“Genetic data, for example short read counts aligned to a reference genome are first pre-processed to give a (phased) variant matrix. Pre-processing of the raw data will typically involve many important analytic decisions (how to impute missing data; choice of thresholds for low read counts or low-quality isolates; what to do with polyclonal samples). The output variant matrix (each row is a phased haplotype, each column is a genetic marker) is then summarised as a distance (similarity) matrix, either using a statistical model or from a direct computation on the variant matrix.”*

And in the next paragraph (why phasing and data pre-processing is outside the scope of this paper):

*“This work addresses the sensitivity of final outputs with respect to the choice of genetic distance and to the algorithm specification. We do not address the important question of sensitivity to pre-processing choices ((this includes allele frequency thresholding, SNP detection algorithms, and imputation of missing values). An important problem in most population genetic epidemiology studies of malaria is what to do with complex infections. There exists software for phasing complex infections which are usually composed of related parasites, e.g. deploidIBD [7], but this is not often done in practice and the implications of ignoring complex infections are not fully understood ( [13] uses simulation to quantify the impact of excluding polyclonal isolates).”*

The reference [13] (Verity et al. *Nature Communications* 2020) is one of the only examples we could find of an applied analysis which specifically did a simulation study analysis to characterise the impact of excluding complex infections.

We completely agree that the PCoA analysis of the -log2IBD matrix is useful. This is what we meant by the statement on line 182:

*“PCoA applied to arbitrary genetic distance matrices is a powerful visual tool, but the interpretation of the output is sensitive to the choice of genetic distance.”*

We have added a sensitivity analysis to the choice of approximation for the upper limit of -log2IBD. This is in the RMarkdown file. The overall structure is preserved but the distance between groups is magnified.

The isolates employed were sympatric and therefore connected or share a recent common ancestor. It is therefore more cautious to interpret PCoA when populations are connected and recombining. Further clarity would have been gained if these three distances were tested for sensitivity against a simulated population with known ancestral recombination graph.

We agree that simulation is necessary but we think that this is a much larger task out of scope of this paper. We are currently applying for funding to do this exact exercise!

I agree with the authors on the arguments against HAC and use of heatmaps for visualisation though these as well were not tested for sensitivity of the seriation approach on distinguishing known clusters (ancestral groups).

This section of the Results (*Visualising distance matrices using heatmaps ordered with HAC algorithms*) is recommends the use of HAC to order distance/similarity matrices as they are empirically very good at finding near-optimal orderings. Our argument is that HAC is a good quick approach to determining initial structure in a distance matrix but should be considered as part of the exploratory data analysis rather than as part of a fully inferential analysis.

We emphasise that this is a useful visualisation approach when we write (lines 257-260):

*“As an illustration, Figure 7 shows heatmaps for the 1-IBD, 1-IBS, and−log2IBD distance matrices, whereby the ordering was determined by HAC with average linkage.”*

Minor issues

- Figure 4 is clearly illustrative of issues with HAC but it is the branch heights that matter and not the horizontal axis which is an arbitrary arrangement.

We agree. The purpose of doing the random permutation shown in Fig 4 was to demonstrate this point exactly. We have extended the paragraph in the Results discussing Fig 4 as although it may be obvious to researchers fluent with tree structures, we believe many people do not understand how sensitive the visualization of meta-data is to the arbitrary choice of leaf ordering.

- Can agglomerative coefficient be used to increase the confidence in HAC

Possibly. However, the value of the agglomerative coefficient is dependent on the number of observations so it is difficult to make recommendations of “good” versus “bad” values. We believe that the best use for dendrograms output from HAC is simply exploratory data analysis. Giving quantitative assessments can be misleading as they are difficult to interpret correctly.

- Testing of HAC as shown in figure 6 gives the impression that the average linking algorithm is best as the stability of its clusters were tested using the other methods. This misinterpretation will remain if either of the other linkage functions were first used.

We agree that this is a limitation of the visualisation. The plot is showing a conditional statement: if you use method A, then how does method B compare? However, this is the best approach to cluster visualisation that we could think of.

We have added to the legend of Fig 6:

*Average linkage was arbitrarily chosen as the `reference' method (any of four linkage functions could be used).*

- The authors gave little explanation on the outputs from figure 7

The purpose of Fig 7 is to illustrate that visualisation of the distance matrix is a useful part of the exploratory data analysis.

- Why did they target only monoclonal isolates? What will happen of populations are highly polyclonal as is the case in some settings in Africa?

This is replied to above (in the major comments).

- The steps before getting the data to use for genetic distance calculation shape the structure of the final set. Recommendations should begin from here.

The purpose of this paper is not to give overall recommendations for how population genetic analyses of malaria parasites should be done. We think this is too ambitious as a goal as multiple approaches could be used, and optimal methods will be highly dependent on contextual analysis goals.

- What are the scientific questions that can be answered by PCoA and HAC?

We do not think that there are (under the currently available theory) specific scientific questions that PCoA and HAC can answer in the context of malaria parasite population genetics. Both these methods could be described as descriptive methods, which allows for the identification of certain features and to rule out other features. For example, these methods are useful to summarise the overall structure of the data. They are of course limited as they are operating on a data summary (pairwise distance).

In the abstract we write:

*“As such, PCoA and HAC can inform (e.g. via exploratory visualisation and hypothesis generation), but not answer comprehensively, key questions about malaria parasite ancestry.”*

Exploratory data analysis (EDA) is a key aspect of all data analytic pipelines. It allows the analyst to better understand complex high dimensional data, identify outliers, and get a general feeling of the stability or robustness of the certain features of the data. But EDA does not allow the analyst to make specific inferential statements. This is what we are trying to say in the abstract and in the paper more generally.

- What are the inferential methods that the 2 approaches can be replaced with?

We are not asking for HAC and PCoA to be “replaced”. As stated in the abstract they *“can inform (e.g. via exploratory data visualisation and hypothesis generation), but not answer comprehensively, key questions about malaria parasite ancestry.”*

A main purpose of the paper is then stated in the abstract: *“we provide tentative guidance for the use and interpretation of PCoA and HAC in malaria parasite genetic epidemiology.”*

This paper serves as a call for the development of better methods:

*“To bridge the inferential disconnect between the output of non-inferential unsupervised learning algorithms and the scientific questions of interest, tailor-made statistical models are needed to infer malaria parasite ancestry. In the absence of such models speculative reasoning should feature only as discussion but not as results.”*

- No proposition of statistical models that can bridge the disconnect they have shown

A stated above, the purpose of this paper is to:

1. Show that HAC and PCoA have been mis-used regularly
2. Give some tentative guidance for how they can be used (they are useful methods)
3. Call for the development of better methods

Methods that fully bridge the disconnect need to be developed. We tried to finish the Discussion on a positive note, highlighting the utility of stopgap methods such as PCoA and HAC:

*By using unsupervised clustering algorithms first (to convert a possibly obscure signal in the data into a summary from which a manageable set of hypotheses can be generated), and statistical models second (to do statistical inference under a manageable set of hypotheses), we can offset the advantages and disadvantages of both unsupervised clustering algorithms and statistical models (fast and versatile but non-inferential versus slow and constrained but inferential) and thus address appropriately the questions of interest.*

- The authors are recommending approaches such as implemented in Relate. How will the problem of differences in recombination rates between populations with different transmission intensities be resolved? While this was not part of the investigation, a recommendation will be useful to the malaria popgen community.

We have not yet had the opportunity to look at Relate in detail. This is clearly a very important new method and deserves to be looked at in detail and tested empirically to see how it compares with other available statistical approaches. We do not feel confident giving recommendations about how to use it at this stage. However, we added in the Discussion (lines 325-326):

*“The issues with HAC algorithms highlighted in this work should be considered when applying Relate to malaria parasite whole genome data.”*

- Emphasis is placed on transparency by making public the distance matrices used. This is an excellent recommendation though it will not resolve issues with published data but rather allow for reproduction or verification

We completely agree. Reproducibility is a very modest requirement! (same data & same analysis -> same results, as per definition in *The Turing Way* <https://the-turing-way.netlify.app/reproducible-research/overview/overview-definitions.html>)

Overall, the paper is timely and highly relevant to the malaria population genetics community.

We thank the reviewer for these detailed comments. We hope that the revised manuscript is improved and clearer.

**Reviewer 2**

I am aware of researchers who have spent a lot of time trying to (unsuccessfully) reproduce Plasmodium falciparum and P. vivax sequence-based population structure analyses and conclusions, including some of those presented within the publications cited in the manuscript. The underlying difficulties in some published work seem to be multi-factorial, including methodologies applied being opaque or non-robust, data being selectively removed, collapsed or not made available (e.g. Indonesian sequence data) and, as per the manuscript, some over-interpretation of results and unawareness of assumptions. Practices are changing and methodologies improving, including with some uptake in the use of IBD approaches, and therefore the proposed manuscript is important for future robust population structure analysis; especially as such analyses could be guiding disease control activities. To strengthen the manuscript, the best practice guidelines therein, and potential impact, I have suggested some edits:

(1) There is a need for additional references in the context of malaria biology or epidemiology. For example, statements like “..unlike humans, malaria parasites can self (recombination between genetically identical male and female gametes), and the rate of selfing varies with transmission intensity.” (line 49) could be supported by a citation. In addition, there is a need for a few sentences discussing different types of machine learning (ML) methods. It seems the manuscript has narrow scope in the ML approaches applied, so some discussion of other approaches that can be used (e.g. random forests, support vector machines, etc. for classification) and examples will be useful for the reader. Perhaps, these approaches be more explicit in Figure 1.

Thank you for these suggestions we have added refs to both statements about malaria biology (refs 6-7 on lines 66-67).

Reviewer 1 suggested removing the reference to machine learning methods, as the methods of interest are more exactly “unsupervised machine learning”. We have changed the title and have removed ML as an abbreviation. Other machine learning methods are very infrequently used in the genetic analyses of malaria parasites so we have focused the work on methods that are routinely used (PCoA, HAC, fineSTRUCTURE). In the Methods we added (lines 503-515):

*“PCoA is the linear version of the more general approach to dimensionality reduction, known as multidimensional scaling. Some analyses of malaria parasite population genetics have used non-linear multidimensional scaling (e.g. [42] uses t-distributed stochastic neighbour embedding [43]) but the advantages of non-linear methods over the linear counterparts are unclear, therefore we consider only PCoA. Supervised learning methods have also been suggested for population genetics [44] but these have been rarely applied to parasite population genetics and are outside the scope of this work. PCoA and HAC are both non-inferential (i.e. they do not have parameters inferred from data via a likelihood function), but we note that some unsupervised learning methods are inferential, e.g. STRUCTURE.”*

(2) One of the issues with analyses of Plasmodium sequencing direct from clinical samples is the multiplicity of infection and potential co-infections (e.g. P. falciparum and malariae co-infections). For the former, the Fws and estMOI software have been applied to triage out samples with >1 clones. It may be worth in the discussion or methods sections (where Fws is mentioned) stating in greater detail how MOI and co-infections can affect genetic clustering and IBD analyses.

We have added in the Introduction lines 127-132:

*“An important problem in most population genetic epidemiology studies of malaria is what to do with complex infections (distinct parasite genomes present in the same human host). There exists software for phasing complex infections which are usually composed of related parasites, e.g. deploidIBD [7], but this is not often done in practice and the implications of ignoring complex infections are not fully understood ([14] uses simulation to quantify the impact of excluding polyclonal isolates). “*

At the start of the Methods section we added (lines 408-417):

*“The data were used to construct matrices of genetic distances between pairs of monoclonal isolates (see next section). Strictly speaking, each ‘monoclonal’ isolate represents a collection of malaria parasites that can be represented by a single parasite genotype. The impact of excluding all infections with complexity above a certain threshold is unclear but has been explored using simulation [13]. In addition, each infection is a subpopulation of parasites with its own ancestry structure. Aggregating this population into a single haplotype (which in fact may not represent any of parasites within the infection) again obscures and possibly biases the estimates of pairwise distance. A better computational pipeline would first phase each infection, and then compute pairwise distances between the n′≥n haplotypes.”*

(3) The starting point for the analyses in the manuscript is a set of SNPs and samples, which have gone through a bioinformatic pipeline. In general, it is extremely difficult to generate exactly the same dataset even with a detailed Methods section. Hence, your call for greater availability of intermediate datasets is a good one. Equally, all underlying raw data needs to be available, and its unavailability is a problem in some P. falciparum and P. vivax genomics studies, where some populations (e.g. Indonesia) appear in print, but their raw data is unavailable. Whilst, I do not want the authors to discuss politics, there is a need for a general sentence about the need for complete data availability and citing some examples of P. falciparum and P. vivax studies where only partial raw sequence datasets were made available.

Data from Indonesia is indeed a good example of the practical limitations faced by journals that wish to enforce fully open data. In the recommendations panel (Panel 2) there is now an additional bullet point:

*“****Publicly available raw data.*** *Full reproducibility implies access to raw primary data (e.g. non-aligned short reads). Laws and regulations of some countries (e.g. Indonesia) make the availability of raw data impossible. However, intermediate data formats, such as VCF, have been used to ensure reproducibility [27].”*

(4) Related to (3), there is a clear need for greater transparency in bioinformatic and statistical/analytical pipelines (this could be included in Panel 1). The effects of allele frequency thresholding, SNP detection algorithms, and how missing genotype values are handled, could also impact on population genetic and structure analyses. It would be worth discussing this briefly.

This is a very important point that was missing from our initial submission. We have added this to Fig 1 (Box 2: *Variant matrix – pre-processing choices*).

We have added in the Introduction (lines 123-127):

*“This work addresses the sensitivity of final outputs with respect to the choice of genetic distance and to the algorithm specification. We do not address the important question of sensitivity to pre-processing choices (this includes allele frequency thresholding, SNP detection algorithms, and imputation of missing values).”*

(5) As stated, there are at least 3 software tools available for IBD analyses. Whilst the focus of the manuscript is not on inter-population comparisons (Fst analyses are cited for this), there is a need to discuss whether and how IBD could be used for such analysis. It is a natural extension from your work to identify the genetic regions that are driving population structure, and by providing a brief discussion of this would lead to greater impact, especially to inform those working on molecular barcodes for geographical and transmission classification. More generally, transmission applications could be referenced.

This is an important question which is not exactly in the scope of this paper but we thought that it deserved proper discussion. We have added a supplementary appendix with the following (S1 Appendix, referenced in the main text on line 447):

*Considering definitions of IBD, this comment touches upon two different points. First, how to transition between inter-individual measures of IBD to inter-population measures. Second, the difference between a per-locus measure of IBD (e.g. IBDsegments as reported in [26]) and a genome-wide measure of IBD (e.g. relatedness as defined in [12]). hmmIBD [25] and isoRelate [26, 51] both generate estimates of IBD segments from the most likely sequence of IBD states; hmmIBD also outputs an estimate of relatedness (“fract_sites_IBD”), while isoRelate could easily be adapted to do so (last time we checked it did not, however). Moreover, it is possible to average over a per-locus IBD measure to generate a genome-wide IBD measure. However, this is not always ideal, especially when the data are limited. For example, a genome-wide measure of relatedness averaged over the most likely sequence of IBD states does not account for all possible sequences of IBD states, which is problematic because the most likely sequence, despite being the most likely, has a very low probability of being the true sequence [52]. Molecular barcodes provide adequate data to estimate inter-individual probabilities of IBD at any position over the genome (e.g. genome-wide measures of relatedness as defined in [12]). They do not provide adequate data to estimate accurately the probability of inter-individual IBD at specified positions over the genome (e.g. IBD segments as reported in [26]). (Aside: whole genome sequence data are required to estimate per-locus measures of inter-individual IBD; these can then be converted into per-locus measures of inter-population IBD, e.g. by averaging over individuals as in [26]) As such, IBD estimates generated from molecular barcode data are not able to identify genetic regions that are driving population structure (e.g. we cannot use molecular barcodes to re-create Fig 4 of [26]). However, they could be used to e.g. monitor changes in transmission (we do not know of an example yet where IBD measures from barcodes have been used to do this; although genome-wide measures derived from WGS data have [53]), and connectivity between locations on a population level (e.g. [29, 30]).For inter-population analyses, one must convert many inter-individual IBD measures into inter-population IBD measures. To date, studies have done this in a variety of ways. For example, by calculating the fraction of inter-individual IBD measures that exceed some threshold (e.g. the fraction whose relatedness is greater than 0.5 [29]). More recently, approaches from optimal transport have been used to in a threshold-free manner that essentially amounts to calculating the cost of transporting a distribution of parasites samples from population to another [30]. There is still a need for a more theoretical analysis of which approach is optimal and in what setting.*

(6) The focus of the manuscript is mainly on SNP analysis, but there are also indel variants, and Figure 1 also mentions microsatellites. Should we include indels to improve population structure resolution and inference? How could indels be included into the ML approaches?

This is a good point. Indels are important in highly clonal parasite populations as they have a higher mutation rate than SNPs (see Redmond et al:

<https://www.ncbi.nlm.nih.gov/pmc/articles/PMC5995194/pdf/msy059.pdf>)

Our understanding is that it is mainly the technological difficulties of correctly sequencing these areas of the genome using Illumina sequencing that limit their use. For example, the README file of the latest Wellcome Sanger Pf 6 data release (the largest openly available set of whole genome sequencing data, <https://www.malariagen.net/resource/26>) states:

*“The VCF files contains [sic] details of 6,051,696 discovered variant genome positions. These variants were discovered amongst all samples from the release. 3,168,721 of these variant positions are SNPs, with the remainder being either short insertion/deletions (indels), or a combination of SNPs and indels. It is important to note that many of these variants are considered low quality. Only the variants for which the FILTER column is set to PASS should be considered of reasonable quality. There are 3,110,990 such PASS variants of which 1,835,719 are SNPs and 1,275,271 indels.”*

However, it then states:

*“Variants are filtered using the VQSLOD metric. VQSLOD is log(p/q) where p is the*

*probability of being true and q is the probability of being false. Theoretically, when VQSLOD > 0, p is greater than q, and therefore the variant is more likely true than false. Conversely, when VQSLOD < 0, the variant is theoretically more like false than true. This is why we have chosen 0 as the threshold to use to declare that variants have passed the filters: all PASS variants are theoretically more likely true than false. Of course, for variants where VQSLOD is only slightly above 0, there is only a slightly greater probability of being true than of being false. Therefore, for example, many of the variants with values between 0 and 1 are likely to be false. Empirically we have found that SNPs tend to be more accurate than indels, coding variants tend to be more accurate than non-coding variants, and bi-allelic variants tend to be more accurate than multi-allelic variants. If you require a very reliable set of variants for genome-wide analysis, and don't mind if you miss some real variants, we recommend using only bi-allelic coding SNPs in the core genome with a VQSLOD score > 6. There are 83,168 such stringent SNPs in the call set. We include a command below to create such a set of variants.”*

Therefore, the use of indels in a population genetics analysis requires understanding their limitations and whether the signal to noise ratio is high enough to warrant their use. In conclusion, we believe that indels are very useful, but with serious technical caveats. We have added on lines 403-407:

*“Only high-quality SNPs from WGS data are routinely used in population genetic analyses due to technical limitations (e.g. see README file https://www.malariagen.net/resource/26). Other polymorphisms, such as indels are useful in highly clonal populations due to their greater mutation rate [40] but currently are not accurately called from short read data.”*

Also, a quick pubmed search revealed many papers performing population STRUCTURE analyses using microsatellites where there is an over-interpretation of “K” (as highlighted, lines 476). Given the large number of studies, it would make sense to include a very recent example to highlight your point and demonstrate that it is a contemporary issue (the most recent one I found from a pubmed search was PMID: 32379762, but feel free to identify an alternative appropriate example).

We have included the most recent example from a pubmed search. In the Discussion we now write:

*This calls into question its applicability to malaria phylogenetics beyond exploratory data analysis (STRUCTURE is often used in the context of microsatellites e.g. [34]).*

It is justified to use this example, as the results section of the abstract of Pacheco et al states:

*Bayesian clustering yields two populations linked to the time of sampling, showing that the parasite populations temporarily changed.*

<https://malariajournal.biomedcentral.com/articles/10.1186/s12936-020-03247-z#citeas>

(7) The dataset used for the analysis is quite small (N=393; covering years 2011-13), especially compared to the much larger datasets currently available. Whilst, I am not suggesting a revised analysis with the Pf3K data from the same locations post-2013, it would be useful to discuss the impact and use of additional data (e.g. for machine "learning"), and any new insights in light of subsequent new control measures.

We used this dataset mainly for illustrative purposes. In some sense it is an “easy” dataset to analyse: small geographic location where samples were collected over a short period of time. This is contrasted to using all available WGS from Southeast Asia (multiple geographic locations, collected over more than a decade). In the latter, explicitly modelling time (allele frequency changes, due to selection and drift) becomes very important. It is unclear how this should be done best.

(8) Please check that gene names are italicised.

We have corrected this in the paper.

**Reviewer #3:**

This is an interesting and thoughtful paper highlighting the lack of critical thinking around the application of ML algorithms in analyses of malaria parasite genetic data.

We thank the reviewer for these comments.

Comments:

I struggled to get a sense of the questions that the authors had in mind in general. In the introduction and discussion there are isolated examples of the questions addressed by the methods. For example, p1 “an important goal in malaria parasite genetic epidemiology is inference of the full ancestral recombination graph” p2 “Many questions of clinical and public health relevance, for example, interpreting reduced haplotype diversity as a selective sweep”, p2 “characterizing population structure”, p13 “construct discrete clusters of samples” and p13 “what is the most likely sequence of events that led to the spread of a single multi-drug resistant P falciparum lineage across Southeast Asia”. There seems to be a swing from hugely broad to very specific questions – I can see why such a problem exists since there are many possible questions. It might be helpful to give some boundaries to the scope of the questions, or to categorize the types of questions, describe the categories and give specific examples within a category.

We agree that this was unclear and so have re-structured the start of the Introduction. The goal of the first paragraph in the Introduction is to motivate the use of genetic distance matrices to characterise population structure (in our particular context for clustering). We now introduce this question by stating:

*“Many of the key questions posed by studies of malaria parasite genetic epidemiology can be phrased in terms of population structure and thus parasite ancestry.”*

All the context specific aspects (what is the most likely sequence of events that led to the spread of a single multi-drug resistant *P falciparum* lineage across Southeast Asia) have now been moved to Panel 1 as you suggested.

There is a long paragraph in the introduction on the epidemic of multi-drug resistant parasites in the GMS. This was interesting, but I felt I had wandered into another paper. It is told from the point of view of someone interested in drug resistance, but for this paper it might be more relevant to focus on the different methods and the different conclusions reached, or to introduce it as a motivating example in a Panel.

This has been moved to Panel 1.

The rationale for the choice of example is not explained. It is interesting, has available data and gives different results by method - but perhaps it is a particular case. *Since the paper is talking in general terms for a wider scope of questions, it may be interesting to consider if there is a set of conditions under which the results for the three methods would be the same.*

These data and analyses of these data (in a larger set of samples collected in the GMS as reported in references 6-8) motivated us to write the paper. We thought that the use and interpretation of HAC and PCoA applied to these data, in the specific context of drug resistance in Southeast Asia could be highly sensitive to the analytic choices. Key outputs of these clustering methods (e.g. the KEL1/PLA1 “lineage”) may not correspond to actual true lineages.

It is unclear how generalisable our results are. We believe that HAC and PCoA have been sufficiently mis-used (in publications in major journals that have considerable impact on public health interventions, e.g. The Lancet Infectious Diseases) to merit a paper that dissects their limitations and advantages. In addition, the new method *Relate* uses HAC in a very specific way. We think it is important to understand the limitations and assumptions of the underlying tree construction method in order to then understand possible limitations of *Relate.* Looking at how *Relate* applies to malaria population genetics is an interesting and useful topic for future work.

Chromosome painting is mentioned several times. It would be helpful to briefly describe how this works.

We thank the reviewer for this suggestion. We have added an explanation of the output of Chromosome painting and we have added the co-ancestry matrix as an example of PCoA (Fig 2).

Lines 493-501:

*As a sensitivity analysis, we compared the PCoA of these three genetic distance matrices with a PCoA of the co-ancestry matrix output from the program fineSTRUCTURE [4]. The co-ancestry matrix is a similarity matrix (increasing values show increasing similarity between pairs). Each entry x_ij_ of the co-ancestry matrix is proportional to the number of ‘chunks’ in the genome donated from isolate j to isolate i. fineSTRUCTURE estimates the co-ancestry matrix with the chromosome painting algorithm. This uses a hidden Markov model to paint each isolate as a piece-wise combination of all other n−1 isolates. A chunk is defined as a contiguous region of the genome unbroken by recombination between i and j.*

There did not seem to be any mention of statistical models to infer networks of infection. Are these outside the scope of the questions considered?

We know of very few analyses in Plasmodium that have used network analysis (if we are understanding the reviewer comment correctly?). A recent example by one of the authors is <https://www.biorxiv.org/content/10.1101/2020.04.10.035303v1> (in Press, PLoS Genetics). We think that network analysis methods (e.g. the stochastic block model) could be exciting approaches for this problem.

We added to the Discussion lines 303-305:

*“As for which methods should be applied to parasite genetic distance matrices, our work illustrates that PCoA is a useful exploratory tool. Other alternatives, such as network analytic methods (e.g. [30]) were not explored in this work.”*

p5 Genetic distance measure 3. It would be helpful to mention how this measure behaves when there is recombination.

We added on lines 491-492

*“When there is recombination for each life-cycle, this distance measure is a linear function of the number of life-cycles.”*

NP - is this written out somewhere? (p12)

We have added the full name for this in the paper (nondeterministic polynomial time)
